# Chromosome-level genome assembly provides new insights into Japanese chestnut (*Castanea crenata*) genomes

**DOI:** 10.3389/fpls.2022.1049253

**Published:** 2022-11-28

**Authors:** Jiawei Wang, Po Hong, Qian Qiao, Dongzi Zhu, Lisi Zhang, Ke Lin, Shan Sun, Shuna Jiang, Bingxue Shen, Shizhong Zhang, Qingzhong Liu

**Affiliations:** ^1^ Shandong Key Laboratory of Fruit Biotechnology Breeding, Shandong Institute of Pomology, Taian, Shandong, China; ^2^ Department of Biology Science and Technology, Taishan University, Taian, Shandong, China; ^3^ State Key Laboratory of Crop Biology, Shandong Agricultural University, Taian, Shandong, China

**Keywords:** japanese chestnut, nanopore, Hi-C, genome sequencing, evolution

## Abstract

Japanese chestnut (*Castanea crenata* Sieb. et Zucc) is an economically and ecologically important chestnut species in East Asia. Here, we presented a high-quality chromosome-level reference genome of the Japanese chestnut cultivar ‘Tsukuba’ by combining Nanopore long reads and Hi-C sequencing. The final assembly has a size of 718.30 Mb and consists of 12 pseudochromosomes ranging from 41.03 to 92.03 Mb, with a BUSCO complete gene percentage of 97.6%. A total of 421.37 Mb repetitive sequences and 46,744 gene models encoding 46,463 proteins were predicted in the genome. Genome evolution analysis showed that Japanese chestnut is closely related to Chinese chestnut and these species shared a common ancestor ~6.5 million years ago. This high-quality Japanese chestnut genome represents an important resource for the chestnut genomics community and will improve our understanding of chestnut biology and evolution.

## Introduction

Chestnut is the only domesticated nut species in family *Fagaceae*, and Japanese chestnut, Chinese chestnut and European chestnut have all been planted for a thousand years in Japan, China and European countries (Conedera and Krebs, 2008; Xing et al., 2019; Nishio et al., 2021). There are four members in the genus *Castanea*, which provide food for humans and other animals, including Chinese chestnut (*Castanea mollissima* Bl.), Japanese chestnut (*C. crenata* Sieb. et Zucc), European chestnut (*C. sativa* Mill.) and American chestnut (*C. dentate* Borkh.). Both Japanese chestnut and Chinese chestnut are blight-resistant, in contrast to European chestnut and American chestnut (Shirasawa et al., 2021).

Japanese chestnut is a woody native plant of Japan and South Korea and is widely cultivated in Asian countries. Similar to Chinese chestnut, several Japanese chestnut cultivars have been selected for their large nut size (Nishio et al., 2021). Compared to Chinese chestnut, Japanese chestnut has larger nuts and better yields but a lower sugar content, and the pellicle is more difficult to remove. However, two newly released cultivars, ‘Porotan’ and ‘Porosuke’, exhibit an easily peeled pellicle, and several studies suggest that this trait may be controlled by one recessive gene (Kurogi and Uritani, 1966; Sato et al., 2007; Sakamoto et al., 2015; Nishio et al., 2021). More research is needed to clone this gene and uncover the molecular mechanism of this important trait in chestnut.

The genome is the foundation of genetic research and has provided many advantages in crop breeding, such as in rice, corn and cotton. Marker-assisted breeding and genomic selection can speed up the breeding cycle, but these methods require one or several high-quality reference genomes. Although seven Chinese chestnut genome assemblies are available publicly (Xing et al., 2019; Staton et al., 2020; Sun et al., 2020; Wang et al., 2020b; Hu et al., 2022), only two Japanese chestnut genome assemblies have recently been released by NCBI (GCA_019972055.1 and GCA_020976635.1); further research is still needed on the genome variations in Japanese chestnut. One of the released Japanese chestnut genomes is for cultivar ‘Ginyose’ and was generated at the scaffold to chromosome-level (Shirasawa et al., 2021); the other is a draft genome assembly that was generated from one 400-years-old native tree in South Korea. Studies have proven that several high-quality genomes help uncover functional genomic variation by direct comparative analysis (Valliyodan et al., 2019; Zhang et al., 2019a; Zhou et al., 2019). Therefore, a high-quality genome and annotation for Japanese chestnut are still needed, and direct comparative analysis between these two genomes may provide useful information for research on this crop.

In this study, Nanopore long-read sequencing and Illumina sequencing were used to assemble the genome of the Japanese chestnut cultivar ‘Tsukuba’, and Hi-C sequencing was used to generate the chromosome-level assembly. Transcriptomes from roots, stems, leaves, flowers and different developmental stages of Japanese chestnut were generated by RNA sequencing and used for genome annotation. Our study provides an opportunity to assess the genome variations between Japanese chestnut and other chestnuts.

## Materials and methods

### DNA extraction and sequencing

Leaf samples from the Japanese chestnut cultivar ‘Tsukuba’ grown in the Germplasm Resources Nursery of Shandong Institute of Pomology, Taian, China, were collected and frozen in liquid nitrogen. Genomic DNA was extracted, size selected and sequenced on an Oxford Nanopore PromethION system by Wuhan Benagen Tech Solutions Company Limited (Wuhan, China). Sequencing adapters were removed from the raw reads, and then low-quality reads were filtered out. For Illumina sequencing, a paired-end library with an insert size of 350 bp was constructed and sequenced by Wuhan Benagen Tech Solutions Company Limited (Wuhan, China) following the manufacturer’s protocol using the Illumina HiSeq X Ten platform.

For Hi-C library construction, fresh young leaves from the same Japanese chestnut tree were fixed using formaldehyde at a concentration of 1%. The chromatin was cross-linked and digested using the restriction enzyme HindIII. The library construction and sequencing followed the method used in our previous research (Wang et al., 2020a).

### Genome assembly and pseudochromosome construction

The primary assembly was generated by NECAT v0.01 and polished by medaka (https://github.com/nanoporetech/medaka) and NextPolish v1.3.1 with the Nanopore reads and Illumina short reads, respectively (Hu et al., 2020; Chen et al., 2021). To anchor the contigs of the primary assembly to the chromosomal-level scaffolds, duplicated contigs were first removed by using Purge Haplotigs v1.1.0 (Roach et al., 2018), and then ALLHiC v0.9.1214 was applied to construct the chromosomal-level scaffolds (Zhang et al., 2019b), Juicer and 3d-dna pipelines were used to adjust and polish the super scaffolds (Durand et al., 2016; Dudchenko et al., 2017). TGS-GapCloser v1.0.1 was used to close sequence gaps in the genome assembly (Xu et al., 2020).

### Genome assembly quality evaluation

Bwa v0.7.17 was used to align the Illumina reads to the primary assembly (Li and Durbin, 2010), and SAMtools v0.1.9 was used to calculate the mapping rate (Danecek et al., 2021). The completeness of the genome assembly was assessed by BUSCO v4.0.2 with the Embryophta_obd10 database (Seppey et al., 2019). LTR_FINDER_parallel was used to detect LTR-RTs in the genome, and LTR_retriever v2.9.0 was used to calculate LAIs for each genome (Ou et al., 2018).

### Repetitive sequence annotation

For repetitive sequence annotation, RepeatModeler v2.0.1 was first applied as the *de novo* method to identify repetitive elements in the genome (Flynn et al., 2020); then, RepeatMasker v4.0.9 was used as the homology-based tool to identify and annotate the repetitive sequences using Dfam v3.151 and Repbase library v20170127 (Zhi et al., 2006).

### Protein-coding gene prediction and functional annotation

The Funannotate v1.7.4 pipeline was used to predict protein-coding genes and functionally annotate the predicted genes (Love et al., 2019). Briefly, RNA-sequencing data were used to train the pipeline first, which employs HISAT2 v2.1.0 (Kim et al., 2019), Trinity v2.8.5 (Grabherr et al., 2011) and PASA v2.4.1 (Haas et al., 2008). Then, multiple gene models were predicted by GeneMark-ES (Lomsadze et al., 2005), Augustus (Hoff and Stanke, 2019), CodingQuarry (Testa et al., 2015), GlimmerHMM (Majoros et al., 2004), SNAP (Korf, 2004) and PASA v2.4.1 by using the training parameters, and EVidenceModeler v1.1.1 (Haas et al., 2008) was used to combine the *ab initio* and evidence-based gene models. The tRNAs were predicted by tRNAscan-SE v2.0.6 (Chan and Lowe, 2019). After filtering out gene models with short lengths (<50 bp), spanning gaps and transposable elements, UTRs were added by the Funannotate update command. Finally, the functions of the proteins were annotated by the EggNOG v4.5.1 (Huerta-Cepas et al., 2017), Pfam v32.0 (El-Gebali et al., 2019), UniProt v2020-08-12 (Apweiler et al., 2004), KEGG (Kanehisa et al., 2014), Gene Ontology (Harris et al., 2004), COG (Koonin et al., 2004), BUSCO v2.0 (Seppey et al., 2019), MEROPS v12.0 (Rawlings et al., 2018), Phobius v1.01 (Kall et al., 2004), SignalP v4.1 (Nielsen, 2017), and CAZyme v8.0 (Lombard et al., 2014) databases or pipelines. We also used KofamKOALA and KofamScan to annotate the proteins (Aramaki et al., 2019) with KEGG Orthologs (KOs).

### Gene family analysis and whole-genome duplication event identification

OrthoFinder v2.5.4 was used to identify gene orthologs and gene duplication events in Japanese chestnut genomes and other genomes (Emms and Kelly, 2019). CAFÉ v4.2.1 was employed to explore gene family size expansion and contraction based on the results of OrthoFinder (De Bie et al., 2006). Whole genome duplication analysis was conducted by using wgd (Zwaenepoel and Van de Peer, 2019), and the Ks distribution of one-to-one orthologs between species was plotted by R packages (ggplot2).

### RNA sequencing analysis and KEGG pathway enrichment analysis

Total RNA was extracted from roots, stems, leaves, flowers, pellicles and three nut developmental stages (70, 80, and 90 days after flowering) of the Japanese chestnut cultivar ‘Tsukuba’. There were three replicates for samples from the different nut developmental stages. The cDNA libraries were constructed and sequenced by Shanghai OE Biotech Co., Ltd. (Shanghai, China) on an Illumina HiSeq 2500 platform. RNA sequencing data were first aligned against the Japanese chestnut genome, and gene counts for each sample were generated by using RASflow (Zhang and Jonassen, 2020). TCC-GUI was used to normalize the expression data and detect the DEGs (Su et al., 2019). TBtools V1.098696 was used to perform the KEGG enrichment analysis based on the KEGG annotation of the proteins (Chen et al., 2020).

### Genome synteny analysis

The Python module jcvi was employed to analyze the synteny between the Japanese chestnut genome, Chinese chestnut genome and oak genome (Tang et al., 2015). SyRI (Synteny and Rearrangement Identifier) was used for structural-variant detection between the two Japanese chestnut genomes (Goel et al., 2019). TBtools was used to show the syntenic genes between different genomes (Chen et al., 2020).

## Results

### Sequencing and assembly of the Japanese chestnut (*Castanea crenata* Sieb. et Zucc) genome

The Japanese chestnut cultivar ‘Tsukuba’ (*Castanea crenata* Sieb. et Zucc) was used for whole-genome sequencing and chromosome-scale assembly. After filtering out low-quality reads, a total of 93.48 Gb of Oxford Nanopore long reads and 80.77 Gb of Illumina short reads were obtained. The sequencing details are provided in **Table S1**. The primary assembly consisted of 469 contigs with a total length of 857.86 Mb and a contig N50 of 5.82 Mb (**Table 1**).

**Table 1:** BUSCO analysis results for the genomes of Japanese chestnut and Chinese chestnut.

Type	*C. crenata*c.v. TsukubaPrimary	*C. crenata*c.v. TsukubaChromosome	*C. crenata*c.v. Ginyose	*C. mollissima*Wang et al	*C. mollissima*Xing et al	*C. mollissima*Sun et al
Total genome length (Mb)	857.86	718.30	721.17	688.93	785.53	773.99
Contig number	469	206	781	652	2707	422
Contig N50 (Mb)	5.82	6.36	1.59	2.83	0.94	5.88
Complete BUSCOs (C)	1579 97.8%	1576 97. 6%	1560 96.6%	1483 91.9%	1578 97.8%	1546 95.8%
Complete and single-copy BUSCOs (S)	1300	1527	1500	1424	1486	1507
Complete and duplicated BUSCOs (D)	279	49	60	59	110	39
Fragmented BUSCOs (F)	13	17	25	34	14	18
Missing BUSCOs (M)	22	21	29	97	22	50
Total BUSCO groups searched				1614		

Three methods were used to assess the quality of the primary assembly. First, the Illumina short reads were mapped back to the primary assembly, and 96.91% (521.82 million reads out of 538.47 million reads) of the total reads were properly paired in the primary assembly, which is significantly higher than several Chinese chestnut varieties (84.24% (‘Yan-Hong’), 90.36% (‘Yan-Shan-Zao-Sheng’), and 89.98% (‘Hei-Shan-Zhai-7’)) (Hu et al., 2022). Second, Benchmarking Universal Single-Copy Orthologs (BUSCOs) analysis showed that 97.8% of the complete BUSCOs were generated in the primary assembly, even though 17.3% of these complete BUSCOs were duplicated (**Table 1**) (Seppey et al., 2019). Third, the LTR assembly index (LAI) score was generated following the method described by Ou et al. (Ou et al., 2018), and the primary assembly yielded a raw LAI = 8.73 and an LAI= 14.36. These results confirmed the high quality of the primary assembly of the Japanese chestnut genome.

**Figure 1:**
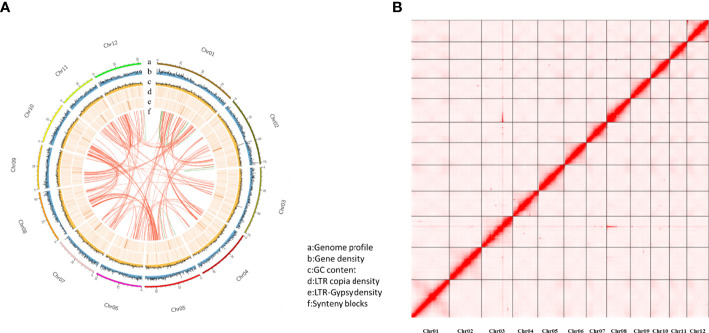
The genome profile and Hi-C contact map for the cultivar ‘Tsukuba’ **(A)** Genome profile; **(B)** Hi-C contact map.

The purged_duplicates assembly consisted of 206 contigs with a total length of 718.30 Mb and a contig N50 of 6.36Mb. BUSCO analysis of the purged_duplicates assembly generated 97.6% complete BUSCOs, with only 3.04% duplicated BUSCOs. Hi-C sequencing generated a total of 302,781,503 read pairs (45.42 Gb). The statistics of the sequencing and mapping details for the Hi-C data are shown in **Table S2**. After mapping the Hi-C reads against the purged_duplicates assembly of Japanese chestnut, 87.11 million valid interaction pairs, accounting for 28.77% of the unique mapped read pairs, were used to construct the chromosomal-level scaffolds. Finally, 12 super-scaffolds were generated, with lengths ranging from 41.03 to 92.03 Mb, accounting for 99.72% of the purged_duplicates assembly. After gap closing by using TGS-GapCloser (Tanaka and Kotobuki, 1992), the final chromosome-scale genome assembly of Japanese chestnut was generated, with fewer 20 gaps per chromosome. The statistics of the pseudochromosomes of the Japanese chestnut genome of the two cultivars are shown in **Table S3**, and the genome profile and Hi-C contact map of the genome of cultivar ‘Tsukuba’ are shown in **Figure 1**.

### Annotation of the Japanese chestnut (*Castanea crenata* Sieb. et Zucc) genome

We used homology-based, *de novo* and RNA-seq methods for protein-coding gene prediction and functional annotation. A total of 46,744 gene models encoding 46,463 proteins were predicted in the Japanese chestnut genome (**Table 2**). A total of 36,074 of 46,463 proteins (77.64%) were annotated by using several databases or pipelines (see methods).

**Table 2:** Genome annotation statics for the Japanese chestnut genome and three Chinese chestnut genome assemblies.

Type	*C. crenata*c.v. Tsukuba	*C. crenata*c.v. Ginyose	*C. mollissima*Wang et al	*C. mollissima*Xing et al	*C. mollissima*Sun et al
Gene number	46,744	69,980	33,612	36,479	45,661
Gene density (per 100 kb)	6.46	10.23	4.87	4.64	5.89
Average gene length (bp)	3880.57	2323.261	4694.974	5091.057	3283.947
Average exon number per gene	5.767	4.535	5.935	5.797	4.904
Average Exon length (bp)	291.70	277.038	295.956	259.149	227.969
Average intron mean length (bp)	881.549	434.626	752.789	1,156.915	790.527
Genome GC content	35.25%	35.14%	35.11%	36.07%	34.99%
Exon GC content	43.30%	44.56%	41.84%	43.36%	43.49%
Complete BUSCOs (C)	1416 87.7%	1223 75.8%	1513 93.8%	1568 97.1%	1481 91.7%
Complete and single-copy BUSCOs (S)	1101	1165	1415	1466	1369
Complete and duplicated BUSCOs (D)	315	58	98	102	112
Fragmented BUSCOs (F)	131	202	33	31	85
Missing BUSCOs (M)	67	189	68	15	48
Total BUSCO groups searched	1614				

A total content of 421.37 Mb of repetitive sequences was annotated in the final assembly of the Japanese chestnut genome, indicating that 58.78% of the genome was repetitive (**Table S4**). Among these repetitive elements, LTR retrotransposons (23.62%) were predominant (14.15% Gypsy, followed by 8.47% Copia), whereas L1 (3.64%) was the most abundant class of LINEs (long interspersed nuclear elements).

### Synteny analysis between the genomes of Japanese chestnut, Chinese chestnut and oak

**Figure 2:**
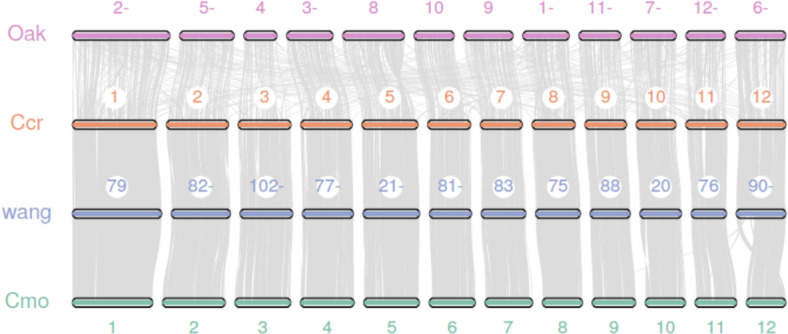
Synteny analysis between the genomes of Japanese chestnut, Chinese chestnut and oak. Oak: *Quercus* spp. Ccr: *Castanea crenata* ‘Tsukuba’, Cmo: *Castanea mollissima* (Sun et al., 2020), and wang: *Castanea mollissima* (Wang et al., 2020b).

We performed a synteny analysis among the genomes of the Japanese chestnut cultivar ‘Tsukuba’, two cultivars of Chinese chestnut (Sun et al., 2020; Wang et al., 2020b) and oak (Plomion et al., 2018). The genome of the Japanese chestnut cultivar ‘Tsukuba’ shows good overall synteny with the two genome assemblies of Chinese chestnut (**Figure 2**). A poor collinearity result was observed between Japanese chestnut and oak. These results might be due to the method used by the previous authors to anchor the contigs to pseudochromosomes in the oak genome; specifically, they used the peach genome as a reference to scaffold the genome (Plomion et al., 2018). For the other two Chinese chestnut genome assemblies, Hi-C sequencing technology was employed to scaffold the genome. This result also suggests the good quality of our genome assembly of Japanese chestnut.

### Structural-variant detection between Japanese chestnut genomes

**Figure 3:**
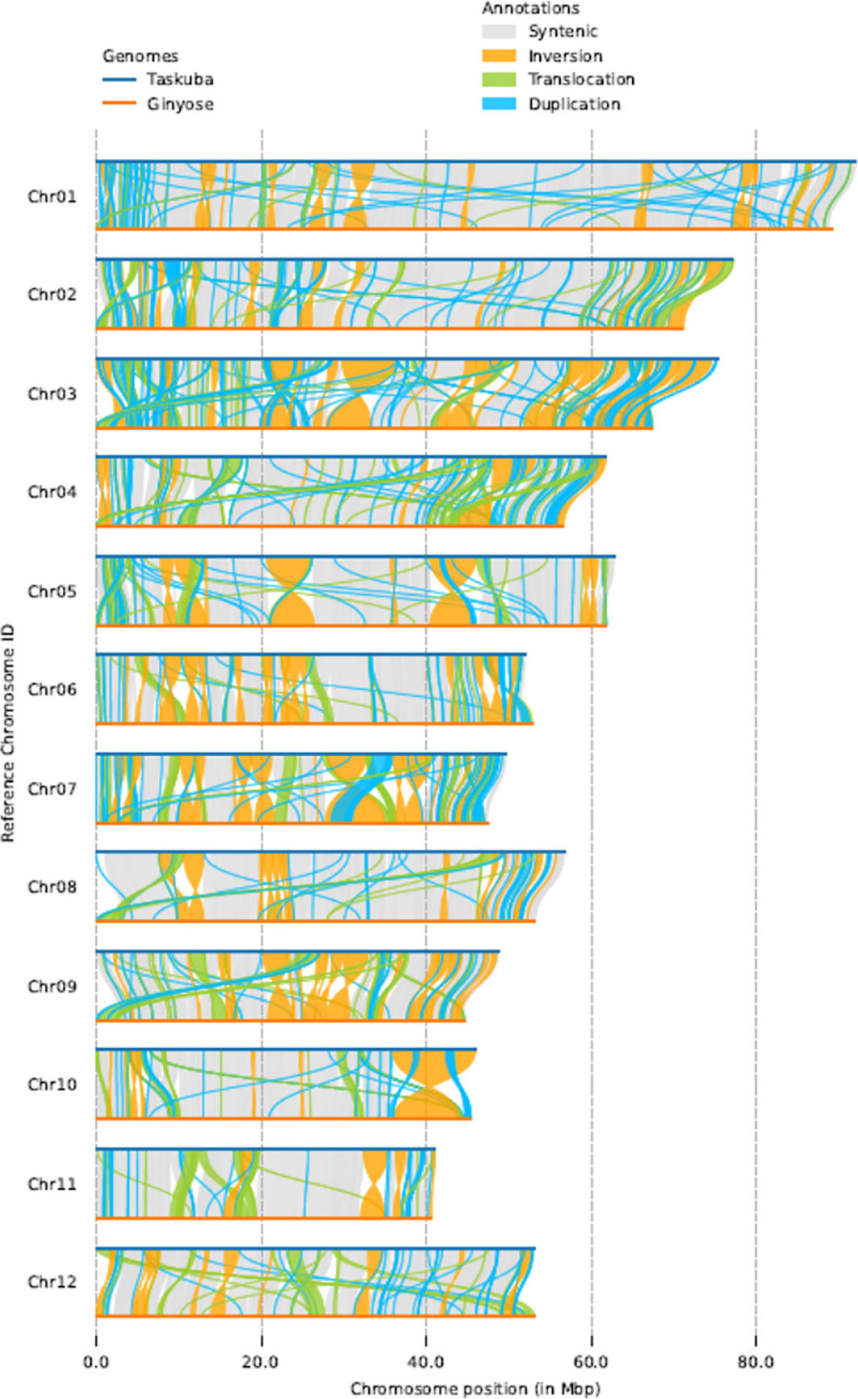
Structural-variant detection between Japanese chestnut genomes.

Using the whole-genome comparison tool SyRI (Goel et al., 2019), we found 414-415 Mb of collinear regions between the genomes of cultivars ‘Tsukuba’ and ‘Ginyose’ (**Figure 3**). In addition, the SyRI analysis identified 207 inversions (accounting for 165.93 Mb in the genome of ‘Tsukuba’), 2,247 translocations (83.27 Mb in the genome of ‘Tsukuba’), and 15,916 duplications in the ‘Tsukuba’ genome (55.65 Mb) and 9,071 duplications in the ‘Ginyose’ genome (37.34 Mb) (**Table S5**). We also identified a 30.12 Mb sequence in the ‘Tsukuba’ genome and a 14.59 Mb sequence in the ‘Ginyose’ genome that were not aligned with each other.

### Evolutionary history and whole-genome duplication events of the Japanese chestnut genome

**Figure 4:**
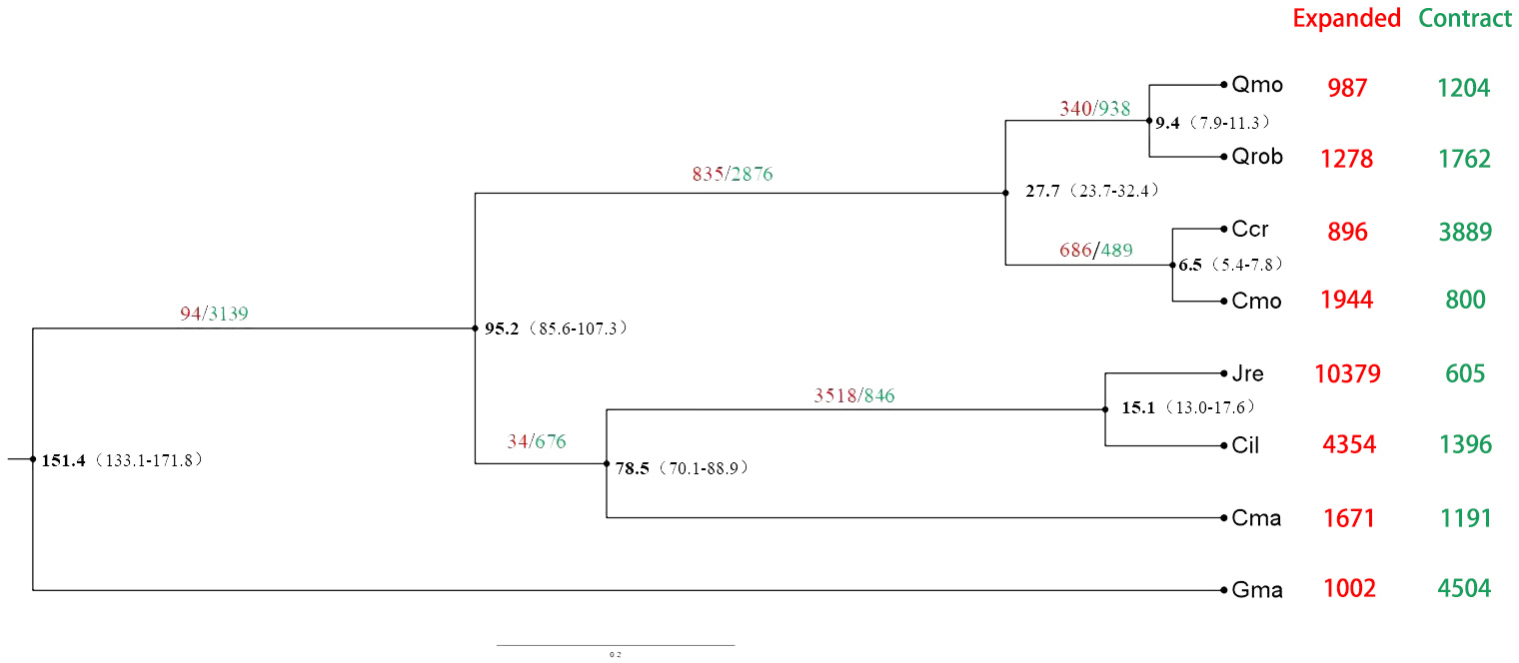
Evolutionary history of the Japanese chestnut genome. Qmo, *Quercus mongolica*; Qrob, *Quercus robur*; Ccr, *Castanea crenata* Sieb. et Zucc; Cmo, *Castanea mollissima*; Jre, *Juglans regia*; Cil, *Carya illinoinensis*; Cma, *Corylus mandshurica*; and Gma, *Glycine max*.

For evolutionary analysis, we selected six other species whose genomes have been sequenced in *Fagales*: three were from the *Fagaceae* family (Cmo=*Castanea mollissima*, Qrob= *Quercus robur*, Qmo= *Quercus mongolica*) (Nakamura, 1994; Plomion et al., 2018; Ai et al., 2022), two were from the *Juglandaceae* family (Jre= *Juglans regia*, Cil*=Carya illinoinensis*) (Neale et al., 2020; Lovell et al., 2021), and one was from the *Betulaceae* family (Cma=*Corylus mandshurica*) (Li et al., 2021). The soybean genome (Gma= *Glycine max*) was used as an outgroup (Valliyodan et al., 2019). A phylogenetic analysis including 346 single-copy orthologous genes confirmed the close relationship between Japanese chestnut and Chinese chestnut (**Figure 4**). Using a reference divergence time of 47-89 million years ago (MYA) between *Fagaceae* and 86-108 MYA between fabids, we estimated that Japanese chestnut and Chinese chestnut shared a common ancestor ~6.5 MYA (**Figure 4**).

**Figure 5:**
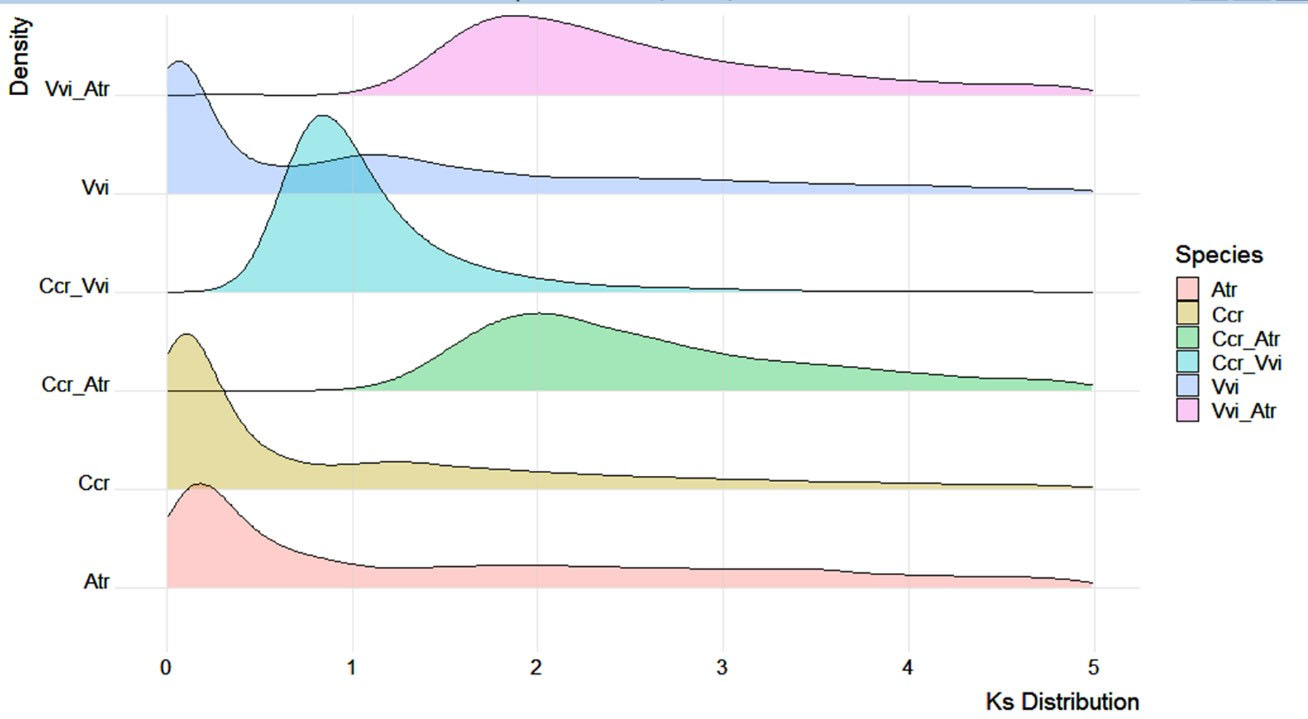
Genome duplication events of the Japanese chestnut genome. Vvi, *Vitis vinifera*; Atr, *Amborella trichopoda*; and Ccr, *Castanea crenata* Sieb. et Zucc.

We calculated the gene *Ks* values in the genomes of Japanese chestnut, grape (*Vitis vinifera*) and Amborella (*Amborella trichopoda*) together with the *Ks* distribution of one-to-one orthologs between these species (**Figure 5**). The Amborella genome is known to be structurally conserved with no lineage-specific genome duplications since angiosperm diversification (Albert et al., 2013), and the grape genome only experienced paleohexaploidization events (Jaillon et al., 2007). We found that the Japanese chestnut genome and grape genome shared a shallow peak at 1.3-1.5, probably reflecting the paleopolyploidy WGD (γ) event in the angiosperm lineage. This result was also confirmed by the Ccr-Atr and Vvi-Atr gene pairs peaking at ~ 2.0. We showed evidence of one WGD event in Japanese chestnut compared with grape, reflected by the Ccr-Vvi gene pair peak at 0.8. These WGD events were also confirmed by the syntenic patterns within the Japanese chestnut genome (**Figure S1**).

### Comparative genomics of gene families

Based on sequence homology, 302,394 proteins accounting for 91.5% of the total genes in these eight species were assigned to 31,300 orthogroups. The statistics for the orthology analysis results are shown in **Table S6**. There were 657 species-specific orthogroups in the Japanese chestnut genome, consisting of 1,929 proteins. The gene duplication event analysis revealed that Japanese chestnut and Chinese chestnut shared 2,078 gene duplication events, whereas only 1,128 gene duplication events were shared by Mongolian oak (*Quercus mongolica*) and pedunculate oak (*Quercus robur* L).

The expansion and contraction of the gene families in Japanese chestnut were analyzed by comparing the gene families within order *Fagales*. There were 987 expanded gene families comprising 3,981 genes and 1,204 contracted gene families comprising 1,377 genes in the Japanese chestnut genome. Japanese chestnut and Chinese chestnut shared 686 expanded gene families and 489 contracted gene families (**Figure 4**).

Of the 987 expanded gene families, 1,609 genes (40.42%) were annotated with KEGG orthology (KO) identifiers and assigned to 421 KEGG pathways (**Table S7**). KEGG enrichment analysis of these genes was conducted, and the ten most significant pathway categories were Vitamin B6 metabolism (17 genes, ko00750), Mismatch repair (25 genes, ko03430), Plant-pathogen interaction (87 genes, ko04626), Glutathione metabolism (44 genes, ko00480), Phenylpropanoid biosynthesis (70 genes, ko00940), Protein export (25 genes, ko03060), DNA replication (23 genes, ko03030), Nucleotide excision repair (23 genes, ko03420), Sesquiterpenoid and triterpenoid biosynthesis (19 genes, ko00909), and Starch and sucrose metabolism (42 genes, ko00500) (**Figure S2**).

In the Vitamin B6 metabolism pathways, 17 genes were identified as pyridoxine 4-dehydrogenase, and there were also 9 genes in the same gene family that were not annotated by the KEGG database. However, only 6 genes in the Chinese chestnut genome were assigned to the same orthogroups, whereas 25 genes in pedunculate oak and 26 genes in Mongolian oak were assigned to these orthogroups. In the Phenylpropanoid biosynthesis pathways, 28 genes in the Japanese chestnut genome were identified as peroxidase and assigned to 5 orthogroups, and the expansion of these genes may be caused by gene duplication events (**Table S8**). Several gene families involved in terpenoid biosynthesis, such as Sesquiterpenoid and triterpenoid biosynthesis and Terpenoid backbone biosynthesis, were also expanded in the Japanese chestnut genome.

In the Starch and sucrose metabolism pathway, 14 proteins were identified as trehalose 6-phosphate phosphatase (TPP) in the Japanese chestnut genome, and 14 proteins in the Chinese chestnut genome were identified as TPP (**Table S9**). However, only 2 proteins in pedunculate oak were identified as TPP, and no TPPs were identified in Mongolian oak. All these TPPs were grouped into one orthogroup in OrthoFinder, and 12 of the 14 TPPs in the Japanese chestnut genome were located in one 450 kb region on Chr11. Synteny analysis of this region between the Japanese chestnut and Chinese chestnut genomes suggested that tandem gene duplication events occurred in these two genomes (**Figure S3**). Since trehalose-6-phosphate (T6P) is a signal of sucrose status and a powerful growth regulator, that coordinates plant growth and development with sucrose supply, TPPs reduced the concentration of T6P to regulate carbon partitioning from source to sink organs (Nuccio et al., 2015; Li et al., 2022). The expansion of these TPPs in the Japanese chestnut and Chinese chestnut genomes might contribute to the higher sugar or starch content in the nut of chestnut compared with that of oak.

### Nut development-related genes in Japanese chestnut

The transcriptomes at three nut development stages (70, 80, and 90 days after flowering) were generated to identify genes involved in nut development in Japanese chestnut. A total of 10,971 genes were identified as differentially expressed genes (DEGs, FDR< 0.05) during nut development. The KEGG pathway enrichment analysis results of these DEGs are shown in **Figure S4** (**Table S10**). Among these pathways, Photosynthesis (Ko00195), beta-Alanine metabolism (Ko00410), Carotenoid biosynthesis (Ko00906), Starch and sucrose metabolism (Ko00500), Pentose phosphate pathway (Ko00030), Flavonoid biosynthesis (Ko00941), Pantothenate and CoA biosynthesis (Ko00770), Limonene and pinene degradation (Ko00903), Linoleic acid metabolism (Ko00591), and Propanoate metabolism (Ko00640) were the ten most enriched metabolic pathways.

Among all these metabolic pathways, Starch and sucrose metabolism had the most gene hits, in which 93 genes were identified as SUS (sucrose synthase [EC: 2.4.1.13]), SS (starch synthase [EC: 2.4.1.21]), GBSS (granule-bound starch synthase [EC: 2.4.1.242]), GPA (glucose-1-phosphate adenylyltransferase [EC: 2.7.7.27]), PGM (phosphoglucomutase [EC: 5.4.2.2]), HK (hexokinase [EC:2.7.1.1]), GPI (glucose-6-phosphate isomerase [EC:5.3.1.9]), GBE (1,4-alpha-glucan branching enzyme [EC:2.4.1.18]), FK (fructokinase [EC:2.7.1.4]), and GPI (glucose-6-phosphate isomerase [EC:5.3.1.9]) (**Table S11**). Most of these genes showed an increased expression level at stage S2 and slight decrease at stage S3, indicating that starch was mainly synthesized at stage S2 in the nut development process of Japanese chestnut (**Table S11**).

Several genes involved in linoleic acid metabolism were also identified as DEGs in the nut development process, which included 11 genes identified as LOX2S (lipoxygenase [EC: 1.13.11.12]), 2 genes identified as LOX1_5 (linoleate 9S-lipoxygenase [EC: 1.13.11.58]), one gene identified as PLA2G (secretory phospholipase A2 [EC: 3.1.1.4]) and one gene identified as TGL4 (TAG lipase/steryl ester hydrolase/phospholipase A2/LPA acyltransferase [EC: 3.1.1.3/3.1. 13/3.1.1.4/2.3.1.51]) (**Table S12**). Most of these genes showed a decreased expression level during stages S1 to S3, and the overall expression level was lower in the nut than in other tissues, which was consistent with the low content of linoleic acid in the nut (**Table S12**).

## Discussion

### Genome assembly quality evaluation of the Japanese chestnut cultivar ‘Tsukuba’

In this study, we generated a *de novo* whole genome assembly of the Japanese chestnut cultivar ‘Tsukuba’ by using Nanopore sequencing and Hi-C technology. The final assembly consisted of 206 contigs with a total length of 718.30 Mb and a contig N50 size of 6.36Mb. Twelve pseudochromosomes were constructed by Hi-C scaffolding, with lengths ranging from 41.03 to 92.03 Mb, accounting for 99.72% of the final assembly.

There is one draft genome assembly of *Castanea crenata* (under accession number GCA_020976635.1) released in NCBI that was generated from one 400-year-old native tree in South Korea. Compared to this genome assembly, our primary assembly shows a longer contig N50 (2.70 Mb vs 6.36 Mb). The BUSCO score of this assembly was also lower than that of our assembly (95.8% vs 97.6%). Since this assembly is only at the scaffold to draft level, and no other information is available, further research is needed when this assembly improves to chromosome-level.

Compared to the genome assembly of the Japanese chestnut cultivar ‘Ginyose’ (Shirasawa et al., 2021), the contig N50 of the genome assembly of cultivar ‘Tsukuba’ improved from 1.59 Mb to 6.36 Mb, and the cumulative length of these two assemblies also confirmed the continuity of the genome of cultivar ‘Tsukuba’ (**Figure S5**). There were also more sequences anchored to the pseudochromosomes in the genome assembly of cultivar ‘Tsukuba’. Ten out of 12 pseudochromosomes in the genome assembly of ‘Tsukuba’ were longer than the genome assembly of ‘Ginyose’ (**Table S3**), which resulted in a total of 34.5 Mb longer sequence in our final genome assembly. There were more complete BUSCOs identified in the genome assembly of cultivar ‘Tsukuba’ than in that of ‘Ginyose’ (97.6% vs. 96.6%) (Shirasawa et al., 2021).

Compared to the two other genome assemblies of Japanese chestnut, our genome assembly of the cultivar ‘Tsukuba’ shows longer continuity and better quality. This high-quality genome may help us identify genes in the Japanese chestnut genome and investigate the domestication and evolutionary histories of Japanese chestnut.

### Annotation and evolutionary history of the Japanese chestnut genome

A total of 46,744 gene models encoding 46,463 proteins were predicted in the genome of cultivar ‘Tsukuba’. However, 69,980 high-confidence genes were predicted in the genome of the cultivar ‘Ginyose’ (Shirasawa et al., 2021). Compared with the former genome annotation of Chinese chestnut (**Table 2**), our genome annotation of Japanese chestnut predicted a similar number of gene models as the research conducted by Sun et al. (Sun et al., 2020) and predicted more gene models than the research performed by Wang et al. (Wang et al., 2020b), Xing et al. (Xing et al., 2019) and Hu et al. (Hu et al., 2022). Compare the BUSCO scores of these predicted gene sets, more than 193 complete BUSCOs were predicted in the Genome of ‘Tsukuba’ than that in the Genome of ‘Ginyose’ (**Table 2**). There were also less missing BUSCOs in the genome of ‘Tsukuba’ than ‘Ginyose’. This result suggested a better annotation of the genome ‘Tsukuba’ than ‘Ginyose’. However, the other three Chinese chestnut genomes generated even better annotations, further research is needed to improve the annotation of the Japanese chestnut genome (**Table 2**).

A total of 421.37 Mb of the sequence was annotated as repetitive sequences in the genome assembly of cultivar ‘Tsukuba’, which was similar to the genome assembly of cultivar ‘Ginyose’ (Shirasawa et al., 2021). The two Japanese chestnut genome assemblies exhibit a repetitive rate similar to that of the genome assembly of Chinese chestnut (431.41 Mb, 55.74%; 437.75 Mb, 64.38%; 423.16 Mb, 53.49%; 442.76 Mb, 64.43%) described by Sun et al. (Sun et al., 2020) and Hu et al. (Hu et al., 2022), but slightly different from that found in research by Wang et al. (366.84 Mb, 53.24%) (Wang et al., 2020b) and Xing et al. (390 Mb, 49.69%) (Xing et al., 2019). This difference might be caused by the exclusion of duplicated contigs in our research or the use of different annotation methods.

Phylogenetic analysis based on single-copy orthologous genes from six other species in Fagales whose genomes have been sequenced, namely, *Castanea mollissima*, *Quercus robur*, *Quercus mongolica*, *Juglans regia*, *Carya illinoinensis*, and *Corylus mandshurica* (Plomion et al., 2018; Neale et al., 2020; Sun et al., 2020; Li et al., 2021; Lovell et al., 2021; Ai et al., 2022), revealed that Japanese chestnut and Chinese chestnut diverged 6.5 MYA.

### Nut development-related genes in Japanese chestnut

Chestnut, as a tree species that has been used to fight against hunger throughout history (Beccaro et al., 2019), should be given more attention and studied. Japanese chestnut is one of the four major chestnut trees in the world. Compared to Chinese chestnut, Japanese chestnut has a larger nut size and better yields, which are advantageous for lessening hunger.

Starch is one of the most important components of chestnuts, and accounts for 50–80% of their dry matter content (Liu et al., 2015). Chestnut starch is considered a potentially functional component of dietary fiber, which may be a source of resistant starch, thus improving health (Liu et al., 2022). Chestnut starch has unique physicochemical properties, such as high swelling power, freeze–thaw stability, pasting viscosity, and low gelatinization temperature (Liu et al., 2015; Liu et al., 2019). In our study, in the Starch and sucrose metabolism pathway, 14 proteins were identified as trehalose 6-phosphate phosphatase (TPP) in the Japanese chestnut genome, and 14 proteins in the Chinese chestnut genome were identified as TPP. However, only 2 proteins in pedunculate oak were identified as TPP, and no TPPs were identified in Mongolian oak (Plomion et al., 2018; Ai et al., 2022). Synteny analysis of this region between the Japanese chestnut and Chinese chestnut genomes suggested that tandem gene duplication events occurred in these two genomes. The expansion of these TPPs in the Japanese chestnut and Chinese chestnut genomes might contribute to the higher sugar or starch content in the nut of chestnut compared with oak (Plomion et al., 2018). Meanwhile, the starch and sucrose metabolism pathway had the most gene hits. Most of these genes showed an increased expression level at 80 days after flowering and slight decrease at 90 days after flowering, indicating that starch was mainly synthesized at 80 days after flowering in the nut development process of Japanese chestnut. Therefore, water and fertilizer management during this period is extremely important.

## Conclusion

In this study, we assembled the genome of the Japanese chestnut cultivar ‘Tsukuba’ by Nanopore long-read sequencing and Illumina sequencing and generated a chromosome-level assembly by Hi-C sequencing. The final assembly had a size of 718.30 Mb and consisted of 12 pseudochromosomes ranging from 41.03 to 92.03 Mb in length, with a BUSCO complete gene percentage of 97.6%. A total of 421.37 Mb of repetitive sequences and 46,744 gene models encoding 46,463 proteins were annotated in the genome. Genome evolution analysis showed that Japanese chestnut is closely related to Chinese chestnut, and they shared a common ancestor ~6.5 million years ago. There were 987 expanded gene families comprising 3,981 genes and 1,204 contracted gene families comprising 1,377 genes in the Japanese chestnut genome. Synteny analysis of this region between the Japanese chestnut and Chinese chestnut genomes suggested that tandem gene duplication events occurred in these two genomes. The expansion of these TPPs in the Japanese chestnut and Chinese chestnut genomes might contribute to the higher sugar or starch content in their nuts of chestnut.

## Data availability statement

The data that support the findings of this study have been deposited into the CNGB Sequence Archive (CNSA) of the China National GeneBank DataBase (CNGBdb) with accession number CNP0003446. Genome assembly and annotation data are available at Figshare: https://doi.org/10.6084/m9.figshare.21391389.

## Author contributions

JW, QQ and QL conducted the experiments and analyzed the data. QQ and PH analyzed the data and prepared the manuscript. DZ, LZ, KL, SS, SJ, BS and SZ performed the collection and processing of samples and analyzed the data. All authors contributed to the article and approved the submitted version.

## Funding

This work was financially supported by the Shandong Provincial Key Laboratory for Fruit Biotechnology Breeding, the Special Fund for Innovation Teams of Fruit Trees in Agricultural Technology System of Shandong Province (SDAIT-06-01), and National Germplasm Repository of Walnut and Chestnut (Tai’an).

## Acknowledgments

We are grateful to the Wuhan Benagen Tech Solutions Company Limited (Wuhan, China) for providing technical support.

## Conflict of interest

The authors declare that the research was conducted in the absence of any commercial or financial relationships that could be construed as a potential conflict of interest.

## Publisher’s note

All claims expressed in this article are solely those of the authors and do not necessarily represent those of their affiliated organizations, or those of the publisher, the editors and the reviewers. Any product that may be evaluated in this article, or claim that may be made by its manufacturer, is not guaranteed or endorsed by the publisher.
