# Supplementary material for: Chromosome-level genome assembly provides new insights into Japanese chestnut (Castanea crenata) genomes
Source: Front Plant Sci. 2022 Nov 28;13:1049253. doi: 10.3389/fpls.2022.1049253 (PMC9742463; doi:10.3389/fpls.2022.1049253)
Supplement: Supplementary file 1 [file DataSheet_1.docx]

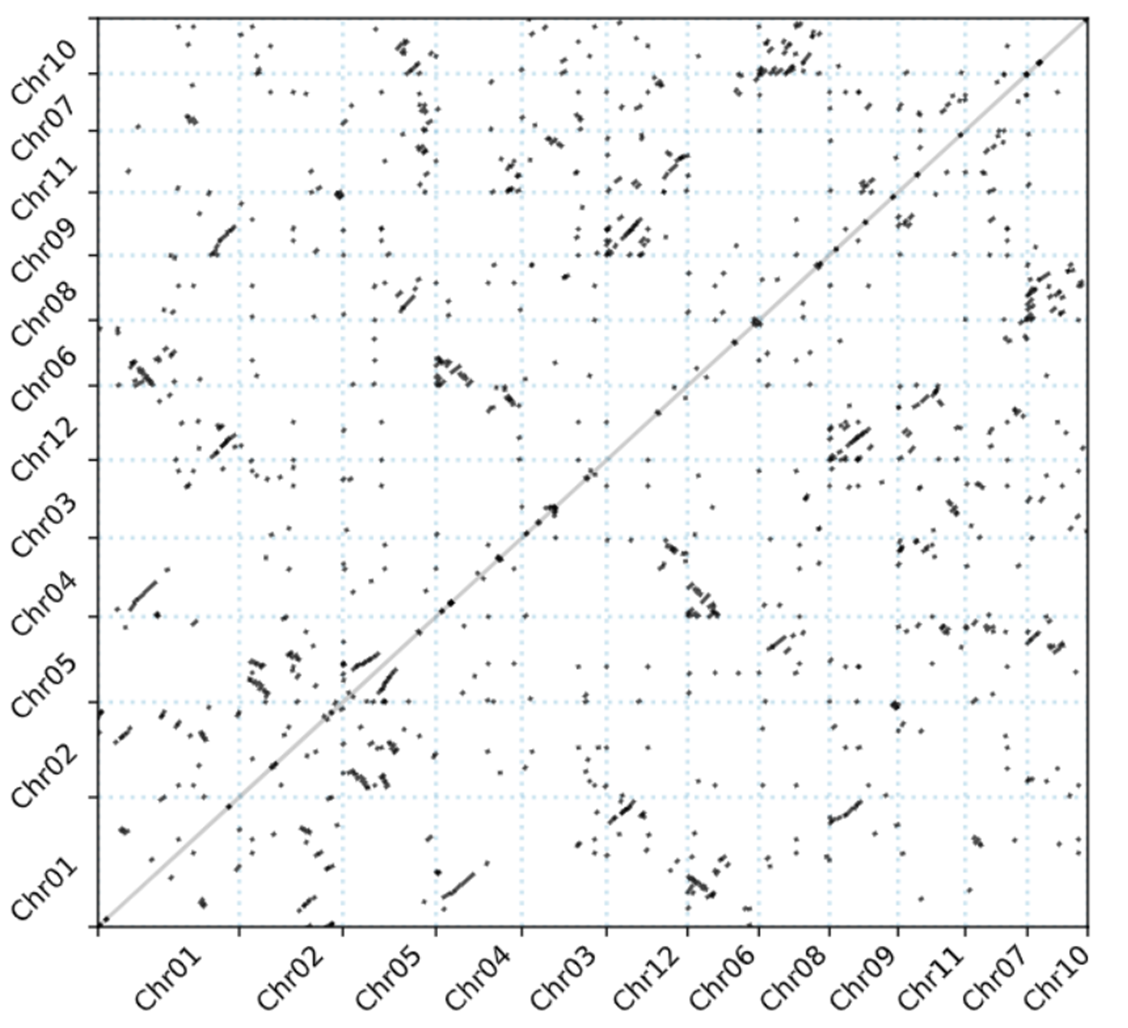


Fig. S1 Wgd-syn analysis results for the genome of the Japanese chestnut cultivar ‘Tsukuba’.


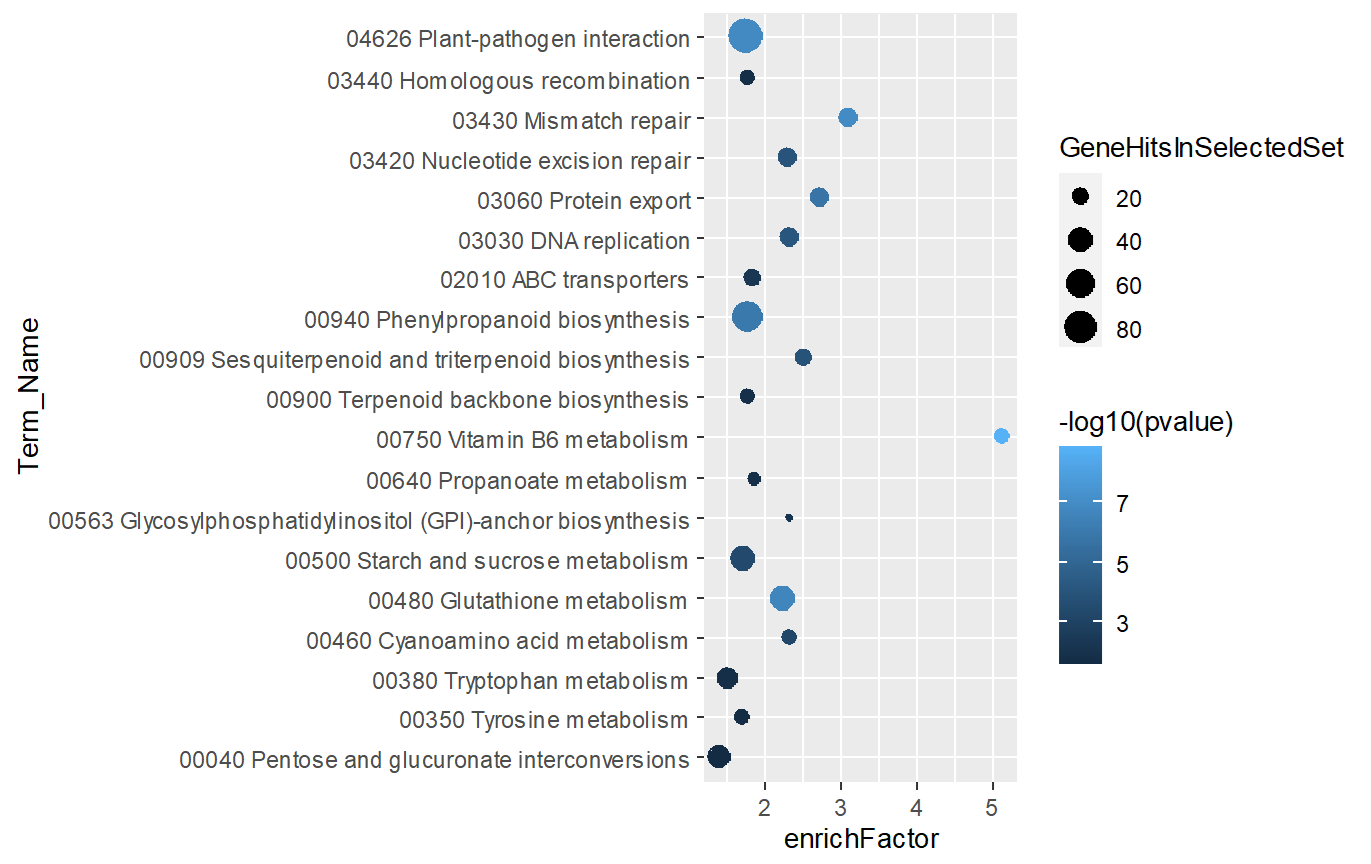


Fig. S2 KEGG enrichment analysis of Japanese chestnut cultivar ‘Tsukuba’ expanded genes.


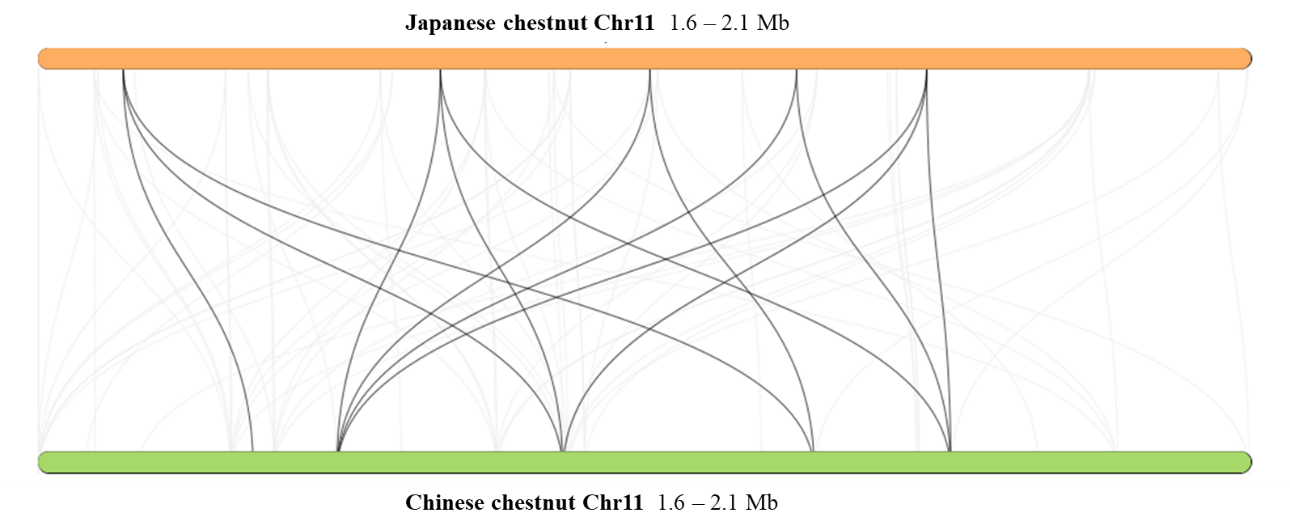


Fig. S3 Collinearity patterns between the Japanese chestnut and Chinese chestnut genomes.


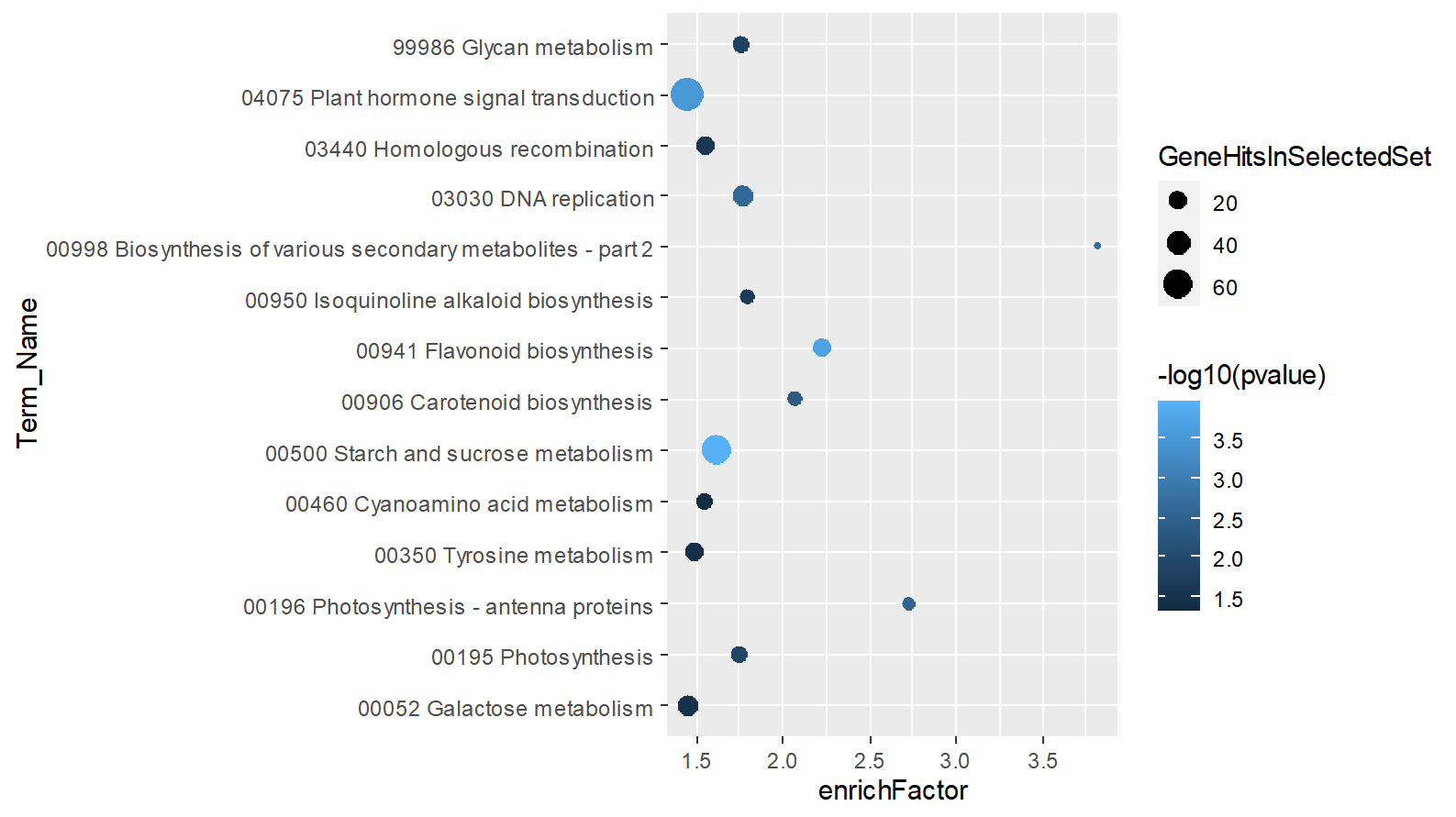


Fig. S4 KEGG enrichment analysis of DEGs involved in Japanese chestnut nut development


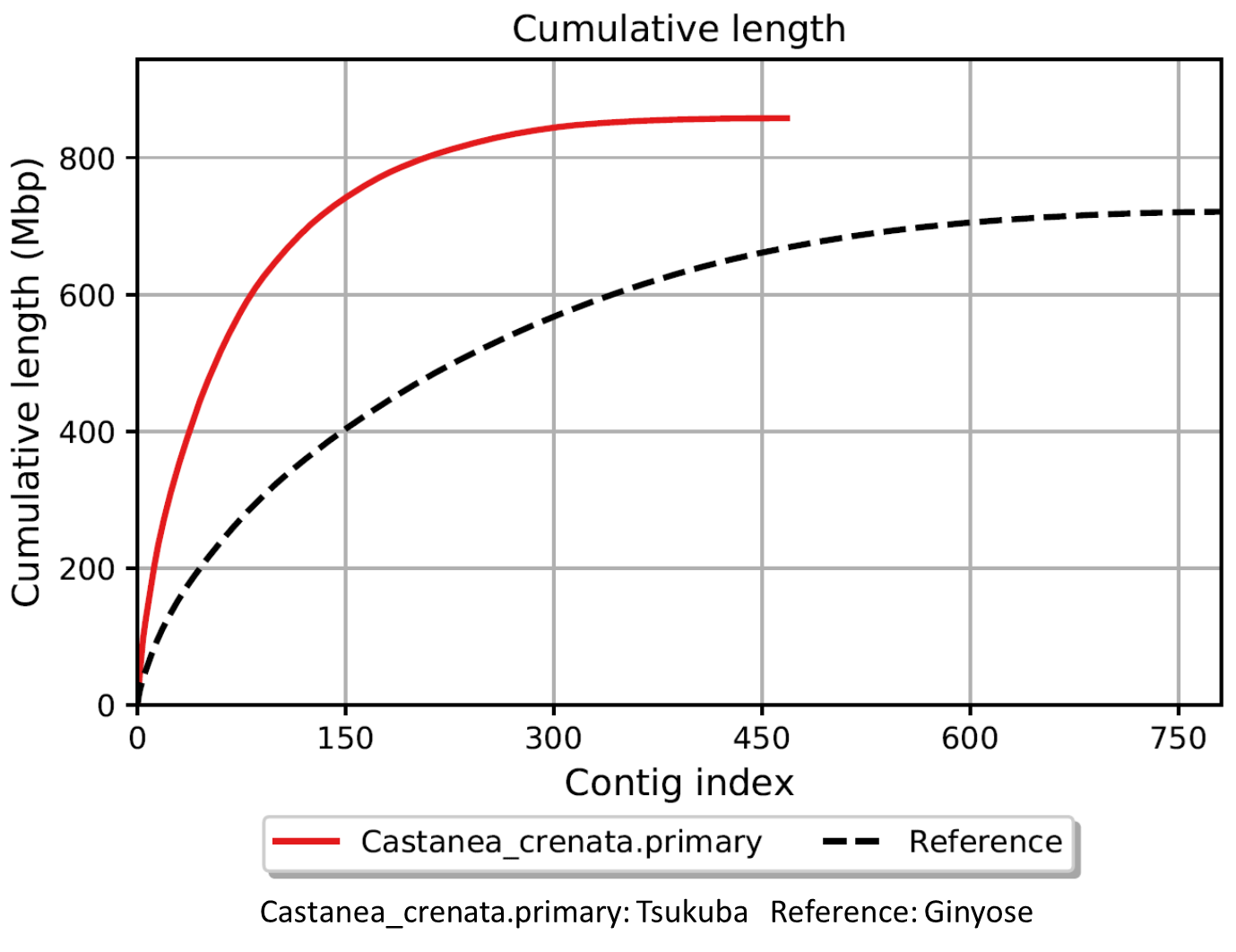


Fig. S5 The genome assemblies of the Japanese chestnut cultivars ‘Tsukuba’ and ‘Ginyose’.
